# Prevalence of Anxiety and Depression among Parents of Children with Cancer—A Preliminary Study

**DOI:** 10.3390/children11101227

**Published:** 2024-10-10

**Authors:** Anna Lewandowska, Tomasz Lewandowski, Anna Bartosiewicz, Katalin Papp, Dana Zrubcová, Mária Šupínová, Aleksandra Stryjkowska-Góra, Barbara Laskowska, Gabriela Joniec, Serap Ejder Apay

**Affiliations:** 1 Faculty of Healthcare, State Academy of Applied Sciences in Jaroslaw, 37-500 Jaroslaw, Poland; barbara.laskowska@pansjar.edu.pl; 2 Faculty of Technical Engineering, State Academy of Applied Sciences in Jaroslaw, 37-500 Jaroslaw, Poland; tom_lew@interia.pl; 3 Institute of Health Sciences, Medical College of Rzeszów University, Rejtana 16 C Street, 35-959 Rzeszow, Poland; abartosiewicz@ur.edu.pl; 4 Faculty of Health, University of Debrecen, 4400 Nyiregyhaza, Hungary; papp.katalin@foh.unideb.hu; 5 Faculty of Social Sciences and Health Care, Constantine the Philosopher University in Nitra, 94974 Nitra, Slovakia; 6 Faculty of Health Sciences, Catholic University in Ružomberok, 03401 Ruzomberok, Slovakia; maria.supinova@szu.sk; 7 Department of Oncology, Radiotherapy and Translational Medicine, University of Rzeszow, 35-959 Rzeszow, Poland; ostryj@o2.pl; 8 Collegium Masoviense, University of Health Sciences in Żyrardów, 96-300 Zyrardow, Poland; joniecgabriela800@gmail.com; 9 Department of Midwifery, Faculty of Health Science, Ataturk University, Erzurum 25240, Turkey; sejder@atauni.edu.tr

**Keywords:** children, cancer, anxiety, depression, parents

## Abstract

Background: A child’s cancer is a highly stressful experience for the entire family. Childhood cancer disrupts family functioning and is one of the most stressful and challenging events parents face, often beyond their control. Parents play a crucial role in providing emotional support to children throughout their illness, and their ability to cope can help reduce the child’s negative emotions. The aim of this study was to assess the prevalence of anxiety and depression among parents of children with cancer. Methods: This cross-sectional study followed the Strengthening the Reporting of Observational Studies in Epidemiology (STROBE) guidelines and included parents of children undergoing cancer treatment. Convenience sampling was used. The Beck Depression Inventory and the Hospital Anxiety and Depression Scale were utilized to assess the parents. Results: This study included 270 participants (73% women, 27% men) with children at an average age of 8.75 ± 4.82 years. Diagnoses included leukemia (53%), lymphoma (29%), and other cancers. On the Beck Depression Inventory, 33% of parents were mildly depressed, 12% moderately depressed, and 32% severely depressed, with an average score of 20.63 ± 12.39 points. The HADS-M scale indicated anxiety at 48.43 ± 20.78%, depression at 45.01 ± 22.8%, and aggression at 54.72 ± 28.71%. Conclusions: Most parents of children with cancer have symptoms of depression and anxiety, which are influenced by the duration of the child’s illness. A strong correlation was observed between the level of anxiety and the tendency for depression.

## 1. Introduction

Despite medical advances that have improved the 5-year survival rate for childhood cancer, cancers remain the third most common cause of death among children [1,2]. The high burden on parents caring for a child affected by cancer, health problems after oncological care, and cancer-related mortality in children negatively affect parents’ mental health [1,2,3,4,5,6,7]. Parents, who play the most important role in pediatric care, are often involved in treatment decisions, constantly monitoring their child’s symptoms and participating in frequent and lengthy hospital visits [7]. It is particularly challenging for parents to face the fear of their child’s potential death, to overcome the conflict between work and caregiving, and to cope with the emotional and practical demands of medical and non-medical care [6,8].

A child’s cancer is a profoundly challenging experience not only for the child and parents but also for other family members [9,10]. The disease disrupts their sense of security, leading to increased anxiety and fear. Childhood cancer significantly impacts family functioning and is one of the most stressful situations a family can encounter, often beyond their control [11]. Recent studies indicate that parents of children with cancer frequently experience heightened levels of stress and anxiety [12]. For instance, a study highlighted that approximately 75% of parents reported noticeable anxiety symptoms, with 87.5% exhibiting a moderate to high risk for post-traumatic stress disorder (PTSD), underscoring the immense emotional burden associated with a child’s cancer diagnosis [13]. Furthermore, research has shown that family members may endure higher stress levels than the cancer patients themselves, with anxiety levels in parents often surpassing those observed in both the affected children and adult cancer patients [14]. The implications of these findings suggest a critical need for support mechanisms to assist families in navigating the psychological challenges posed by childhood cancer.

Coping with a child’s illness is challenging, which is why many parents suffer from anxiety and depressive disorders and even post-traumatic stress disorder [15]. Numerous studies have shown that compared to the general population, parents of children with cancer often experienced post-traumatic stress, uncertainty, anxiety, and depression during the first year of treatment, and poorer mental health of parents can persist for many years after treatment [8]. As reported in the global literature, the prevalence of clinically significant anxiety and depression among parents of children with cancer is notably high, with rates of 74% and 46%, respectively [1,15,16]. In contrast to previous studies, including those by Mess et al. [15] and Seiler et al., which primarily explore the psychological impacts of cancer on patients and their families, our study focuses specifically on the role of perceived social support in mitigating anxiety and depression among parents of children diagnosed with cancer [17]. Mess et al. investigate the emotional struggles faced by families coping with cancer—particularly the prevalence of anxiety and depression [15]. A study by Khoury and Egozi supports this finding, highlighting that parents of children with cancer experience significant psychological distress, with high rates of anxiety and depression during the treatment period. This research emphasizes the persistent mental health challenges that can arise not only during treatment but also in the following years [12]. A descriptive study conducted by Feki et al. indicates that the prevalence of anxiety and depressive disorders is high among parents of children diagnosed with cancer. They found a significant correlation between stress and anxiety and the female sex in parents. Sixteen percent of the parents had scores indicating acute stress, while twenty-one percent had scores suggesting post-traumatic stress. Eighty-six percent of the parents experienced mild to severe depression, and ninety-five percent had minor to major anxiety. Furthermore, post-traumatic stress and anxiety are significantly correlated with the female sex in parents, and significant correlations were also found between post-traumatic stress scores and symptoms of depression and anxiety [18].

In Ethiopia, parents of children with cancer identified several primary stressors: the severity of their child’s condition, fears regarding unfavorable treatment outcomes, limited access to cancer treatment services, and insufficient social and financial support. To alleviate parental stress, several strategies could be implemented, such as offering psycho-oncological support for parents and enhancing counseling related to their child’s illness, treatment options, diagnostic processes, and potential side effects of treatment. Additionally, establishing and bolstering family support networks, facilitating communication among parents, improving the accessibility of chemotherapy medications, and providing education on coping mechanisms could also be beneficial [19]. Moreover, many studies highlight that parental mental health often deteriorates in response to their child’s diagnosis, with a notable prevalence of post-traumatic stress symptoms. A qualitative exploratory study in Ghana emphasizes coping strategies adapted by parents caring for children with cancer and advocates for tailored interventions aimed at enhancing the psychological resilience of parents. This aligns with Mess et al.’s [15] focus on the need for comprehensive support systems [20].

These studies collectively underscore the pressing need for targeted psychological support for parents of children with cancer, reflecting a growing recognition of their mental health needs within the context of pediatric oncology [11,12,17,18,19,20].

Based on the analyzed studies, we can formulate the hypothesis that higher levels of perceived social support are associated with lower levels of anxiety and depression among parents of children diagnosed with cancer. This hypothesis can be tested by analyzing the relationship between perceived social support (measured through surveys or questionnaires) and mental health outcomes (specifically anxiety and depression levels) among the participants [21,22].

Parents play a crucial role as the primary source of emotional support for children with cancer throughout the various stages of the disease, as shown in various studies that highlight their importance in shaping a child’s emotional resilience during treatment [23,24]. Their attitudes and coping abilities can significantly influence how children experience negative emotions associated with the diagnosis and treatment [24,25], demonstrating the profound impact parental mental health has on their child’s emotional adjustment [23]. Research indicates that when parents experience high levels of stress, anxiety, and depression, it can exacerbate their child’s anxiety, creating a cycle of emotional distress that affects the entire family [23,26]. Understanding the complexities of the different treatment phases and the overall family dynamics is essential for providing comprehensive psychological support. Parental suffering can profoundly impact a child’s ability to adapt to their illness, their recovery process, and their overall functioning [24]. Thus, addressing the emotional needs of parents is not only beneficial for them but also critical for the well-being of their children during this challenging time.

The aim of this study was to assess the prevalence of anxiety and depression among parents of children with cancer. 

## 2. Materials and Methods

### 2.1. Study Design

This cross-sectional study was conducted in 2024 according to the Strengthening the Reporting of Observational Studies in Epidemiology (STROBE) guidelines [27,28].

The selection of a cross-sectional study design is particularly advantageous in exploring the prevalence and associations of health-related variables at a specific point in time. In recent studies, the cross-sectional design has been employed effectively in similar research contexts. For instance, Thompson et al. utilized a cross-sectional study to examine the mental health outcomes of caregivers in pediatric oncology, identifying significant relationships between caregiver stress and child health outcomes. This design facilitated the collection of data from a diverse sample of caregivers, allowing for robust statistical analyses of the factors influencing their psychological well-being [25]. Another example is provided by Mezgebu et al., who conducted a cross-sectional study assessing predictors of resilience among parents of children with cancer. Their findings underscore the utility of cross-sectional studies in identifying urgent needs for support services in clinical settings [29].

### 2.2. Study Participants

A convenience sampling method was utilized due to the limited availability of study participants and the need to collect data within a shorter time frame. Previous research indicates that this method can be effective in obtaining representative results, particularly in studies involving hard-to-reach populations, such as those affected by pediatric cancer [14,30,31,32].

This study was conducted among parents of children with cancer undergoing treatment. Inclusion criteria for this study were the participants’ consent to participate in this study, consent to complete the questionnaire, and the child’s cancer being under treatment. Exclusion criteria were the lack of consent to participate in this study and the lack of consent to complete the questionnaire. After obtaining permission from the administrator of the OnkoParents forum to conduct this study among forum users, an electronic version of the questionnaire along with a declaration of voluntary participation was posted on the website. Due to this being a pilot study, a total of 206 participants who correctly completed the questionnaire were included in the analysis. There was no direct incentive to participate in this study; however, the importance of the issue—understanding the psychological impact on parents of children with cancer—likely motivated participants to contribute.

### 2.3. Instruments

This study used a questionnaire survey, the Beck Depression Inventory, and the Hospital Anxiety and Depression Scale (HADS-M).

The questionnaire includes detailed and extensive instructions on how to complete it. It contains open-ended, single-choice, and multiple-choice questions, allowing for the collection of demographic, epidemiological, and qualitative information. The questionnaire was divided into a general part and a detailed part. To better understand the context of this study and the analysis of the results, while also identifying potential confounding variables that may influence this study’s outcomes [33], the demographic section of the questionnaire includes information such as gender, age, parental education, and place of residence.

The Beck Depression Inventory (BDI) is a self-assessment tool used for the preliminary diagnosis of depression. It consists of 21 questions that need to be answered. For each question, one response that best represents feelings over the past 7 days should be selected. Responses are scored from 0 to 3 points. After completing the questionnaire, the results should be interpreted. Scores from 0 to 11 indicate no depression, suggesting a temporary mood deterioration. Scores from 12 to 19 indicate mild depression, suggesting the need to consult a psychologist for diagnosis. Scores from 20 to 25 indicate moderate depression, recommending prompt contact with a psychologist/psychotherapist or psychiatrist. Scores from 26 to 63 indicate severe depression, suggesting a dangerous health and life condition. A visit to a psychiatrist is recommended. Selected cases may require hospital treatment to prevent life-threatening situations. The psychometric properties of the Beck Depression Inventory (BDI) are as follows: it demonstrates high internal consistency across various populations, typically with Cronbach’s alpha coefficients ranging from 0.85 to 0.95. The BDI also shows good test–retest reliability, with correlation coefficients often exceeding 0.90 when administered to the same subjects over a short period. Factor analyses of the BDI generally support its construct validity, confirming that it primarily measures symptoms of depression [34,35,36].

The Hospital Anxiety and Depression Scale (HADS) is used to detect anxiety and depressive disorders. Clinical diagnosis cannot be based on its result, but it can detect the occurrence of low mood and anxiety. The HADS scale is also used to assess the severity of anxiety and depression. This study used the Polish version of the HADS scale (HADS-M), which consists of 16 statements. The respondent answers on a four-point scale. Each statement on the scale is rated from 0 to 3 points. For both anxiety and depression, 7 points is accepted as the cut-off threshold. The HADS-M scale assesses the severity of anxiety, depression, and aggression in the respondents. The results of the scale are presented in points and as a percentage of the maximum possible points due to the fact that the maximum number of points to be obtained is not equal between the three categories.

The HADS scale demonstrates good internal consistency, with Cronbach’s alpha values typically ranging from 0.78 to 0.93 for both the anxiety and depression subscales. This indicates that the items within each subscale effectively measure a cohesive construct. Additionally, the scale exhibits strong test–retest reliability, with correlation coefficients generally exceeding 0.80 when administered to the same subjects over a short period. Factor analyses have confirmed the two-factor structure of the HADS, effectively separating anxiety and depression symptoms. This supports its construct validity, as it reliably measures distinct but related constructs [37,38,39].

### 2.4. Data Collection

This study was conducted online among parents of children with cancer. An electronic version of the questionnaire was created using Google Forms (https://forumonkologiczne.pl/forum/onkologia-forum-dla-rodziny-i-pacjenta/749/watki/1, access date December 2023). The questionnaire was posted on the OnkoParents internet forum [40], with a request for parents of children with cancer to complete it. Each respondent was informed about the purpose and course of this study and about the possibility of refusing to participate or withdrawing from this study at any time. Respondents were instructed on how to complete the questionnaire. The questionnaire includes single-choice, multiple-choice and open-ended questions. The prepared research tool was verified to check how well it measures the phenomenon we want to understand. The pilot study to verify and standardize the questionnaire was conducted on a small sample group of 30 people, checking whether all questions were clear and understandable to the respondents, whether they were understood according to the researcher’s intention, and whether they provided the information the researcher wanted to obtain.

### 2.5. Sample

The survey was conducted among parents from OnkoParents Forum. This is an online platform specifically designed for parents of children diagnosed with cancer. It serves as a vital support network where parents can share their experiences, seek advice, and connect with others facing similar challenges. This forum fosters a sense of community among users who understand the emotional and practical struggles involved in caring for a child with cancer. Members often share valuable resources, such as articles, medical information, and links to support organizations. This helps parents access important information about treatment options, coping strategies, and available services. The forum also allows for anonymity, enabling parents to discuss sensitive topics without fear of judgment. In addition to medical information, the platform offers guidance on psychological, social, and therapeutic support for both sick children and their caregivers, making it a holistic resource. The OnkoParents Forum may also host events, workshops, or initiatives aimed at raising awareness about childhood cancer and supporting affected families.

The forum was established through the initiative of the ISKIERKA Foundation, in collaboration with medical professionals and technology companies, including 3Soft (Katowice, Poland) and Netology (Katowice, Poland). The involvement of the Polish Society of Pediatric Oncology and Hematology as the editorial supervisor further emphasizes the forum’s commitment to offering credible and evidence-based information [40]. The OnkoParents Forum serves as a vital resource for Polish-speaking parents of children diagnosed with cancer, offering tailored support that addresses the specific challenges they face within the Polish healthcare system [27]. In contrast, platforms like St. Jude’s Children’s Research Hospital’s family support forums also provide various events and workshops, but these tend to have a more generalized or international scope, focusing on broader issues rather than local healthcare challenges [41]. While such platforms are beneficial in offering comprehensive guidance, they may not fully address the nuanced needs of families dealing with pediatric cancer in Poland. Furthermore, platforms such as CancerCare (New York, NY, USA) for Kids maintain strong ties to medical communities, offering access to licensed oncology social workers and healthcare professionals. However, the collaboration between the OnkoParents Forum [40] and local experts provides parents with direct access to country-specific medical insights, ensuring that the support they receive is both relevant and actionable within their specific context [42].

This tailored approach not only enhances the relevance of the information provided but also fosters a strong sense of community among users, enabling them to share experiences and support one another effectively [43].

This study involved 206 participants, including 73% women and 27% men, 62% of whom were married. Most respondents had higher education (56.6%), and 57% were urban residents, while 43% were rural residents.

### 2.6. Ethical Considerations

This study was approved by the Bioethics Committee at Collegium Masoviense University of Health Sciences in Zyrardow no. 51/2023 (20 December 2023). Participation in this study was voluntary and anonymous and respondents were informed of their right to refuse or withdraw from this study at any time. Each participant was informed about the purpose of this study and the time of completion of this study.

### 2.7. Data Analysis

Statistical analysis of the collected material was conducted using the software Statistica 13.3 (TIBCO Software Inc., Palo Alto, CA, USA), a comprehensive tool widely used in scientific research for data analysis. Statistica 13.3 offers robust non-parametric test options, ideal for situations where data do not meet parametric assumptions, such as normal distribution. This software has been employed in various studies to ensure accuracy and validity in handling non-normally distributed variables [44].

In this study, the Shapiro–Wilk test was utilized to assess the normality of distributions. The Shapiro–Wilk test is a well-established method for detecting deviations from normality and is particularly suitable for small to medium sample sizes [45].

Descriptive statistics were used as a fundamental part of data analysis, providing essential summaries of the sample and the variables being studied. In this study, descriptive statistics were used to calculate key parameters for numerical variables, such as the mean (representing the average value of the variable), median (indicating the middle value in a data set, less sensitive to outliers), minimum and maximum values (showing the range of the data by identifying the smallest and largest values), first and third quartiles (highlighting the spread of the data and where most of the data lies, with the 25th percentile for the first quartile and the 75th percentile for the third), and standard deviation (measuring the amount of variation or dispersion in the data.

The following non-parametric tests were used:

- The Mann–Whitney U test was employed to compare differences between two independent groups. This test is widely used when the assumption of normal distribution is violated and is the non-parametric alternative to the independent *t*-test [46].
- Spearman’s rank correlation coefficient was used to evaluate the strength and direction of association between two continuous variables that do not follow a normal distribution. This coefficient is often cited as the non-parametric counterpart to Pearson’s correlation [47].
- A one-sided structure index significance test was applied to analyze differences in categorical data (e.g., percentages of responses). This test allows for hypothesis testing regarding proportions and is often used in binomial data analysis [48].

A significance level of *p* < 0.05 was set for all statistical tests, a standard threshold in psychological and medical research to denote statistical significance.

## 3. Results

### 3.1. Demographic Data of the Studied Groups

The studied group consisted of 206 respondents, including 73% women and 27% men. Among the respondents, 69% were professionally active. Other descriptive statistics identifying the studied group are presented in Table 1.

### 3.2. Family Characteristics of the Studied Group

**Figure 1:**
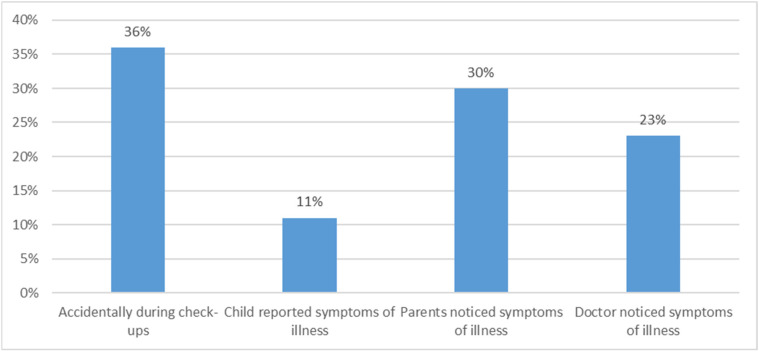
Circumstances of cancer diagnosis in the children in this study.

**Figure 2:**
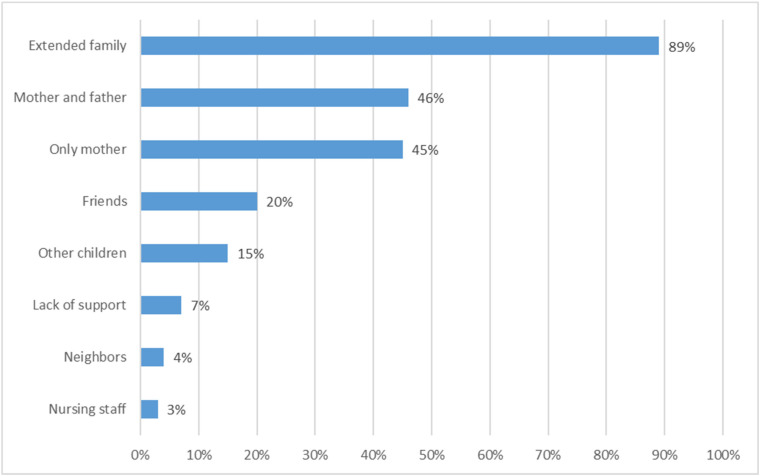
Support in childcare.

Among the children, 43% were girls and 57% were boys. The children of the surveyed parents were aged from 1 to 22 years. The average age of the studied children was 8.75 years ± 4.82 years. Among the children, 53% had leukemia, 29% had lymphoma, 7% had central nervous system cancer, 6% had neuroblastoma, 4% had Wilms’ tumor, and 1% had glioma. A total of 19% of the children had been battling cancer for up to 3 months, 24% for 3 to 6 months, 31% for 6 to 12 months, and 26% for over 12 months. The time elapsed since the start of treatment was up to 1 month for 15%, 1 to 3 months for 17%, 3 to 6 months for 15%, 6 to 12 months for 30%, and over 12 months for 23%. A total of 28% of the children underwent radiotherapy, 89% chemotherapy, 3% hormone therapy, and 25% were treated surgically. Cancer was detected in the studied children under various circumstances, but most often accidentally during check-ups (36%) (Figure 1). More than half of the children included in this study were currently in the hospital (52%), 38% at home, and another 10% in hospice. The main caregiver for the sick child was only the mother (45%), in 46% both the mother and father, and in 3% it was only the nursing staff (Figure 2).

### 3.3. Psychological State of the Studied Group

On the Beck Depression Inventory, respondents scored an average of 20.63 points ± 12.39 points, with a minimum of 0 and a maximum of 63 points. Interpreting the Beck Inventory results, 33% had mild depression, 12% moderate depression, and 32% severe depression. A total of 23% of respondents had no symptoms of depression. It was confirmed that significantly more parents had symptoms indicating depression (77%). This difference was statistically significant (*p* < 0.001) (Table 2).

The HADS-M scale assessed the severity of anxiety, depression, and aggression in the respondents. The results of the scale were presented in points and as a percentage of the maximum possible points due to the fact that the maximum number of points to be obtained was not equal in the three categories. On the percentage scale, the severity of anxiety in the studied parents was assessed at an average of 48.43 ± 20.78%, depression at an average of 45.01 ± 22.8%, and aggression at an average of 54.72 ± 28.71% (Table 3).

Significant statistical relationships were found between the severity of depressive symptoms in the studied parents and the level of anxiety and aggression they experienced. The results obtained on the Beck Depression Inventory correlated with the results obtained on the HADS-M scale in the category assessing the severity of depressive symptoms (R = 0.68 *p* < 0.001). It was also shown that a greater severity of depressive symptoms was significantly associated with a greater severity of anxiety (*p* = 0.001) and aggression (*p* < 0.001), with positive values of R = 0.31 and R = 0.58, respectively. No differences were found between the severity of depressive, anxiety, and aggressive symptoms among parents under 40 years of age and those aged 40 and over (*p* > 0.05). No differences were also found between the severity of depressive, anxiety, and aggressive symptoms among the studied women and men (*p* > 0.05). The level of aggression in the studied parents did not depend on their level of education (*p* = 0.495). However, it was shown that parents with lower education levels experienced a greater severity of anxiety (*p* = 0.019) and depression (*p* < 0.001) compared to parents with higher education levels. The severity of depressive, anxiety, and aggressive symptoms in parents was not dependent on the age of their children (*p* > 0.05). The level of depression among parents of children with cancer did not depend on the duration of the child’s illness. However, the symptoms of anxiety and aggression among parents differed significantly depending on the duration of the illness. It was shown that parents of children who had been ill for longer (more than six months) experienced a greater severity of anxiety (*p* = 0.001) and aggression (*p* = 0.012) (Table 4 and Table 5).

## 4. Discussion

The assumptions of the hypothesis that higher levels of perceived social support are associated with lower levels of anxiety and depression among parents of children diagnosed with cancer are confirmed in the scientific literature. Numerous studies indicate that parents who perceive greater social support experience significantly reduced psychological distress, including anxiety and depression [12,18]. This correlation underscores the importance of social support systems in mitigating the emotional burdens faced by parents during their child’s cancer journey. Furthermore, effective coping mechanisms linked to strong social networks can enhance parental resilience and emotional well-being, reinforcing the need for targeted interventions that foster these support systems [19,20]. Recent studies highlight the emotional challenges faced by parents, indicating that many experience significant psychological distress throughout their child’s treatment journey. The studies collectively underscore the emotional toll that caring for a child with cancer can have on parents, emphasizing the need for tailored psychological support to help them cope with their experiences [49,50,51]. Similarly, other researchers noted that parental stress levels can be exceptionally high, driven by fears about their child’s prognosis and the uncertainties of treatment [52,53].

Our findings corroborate these insights, revealing that a substantial proportion of parents in our study exhibit symptoms of anxiety and depression. Specifically, we noted that 77% of parents reported experiencing depressive symptoms, consistent with the prevalence rates identified in the meta-analysis conducted by van Warmerdam et al., which highlighted the heterogeneity of anxiety and depression rates among this population [54]. This underscores the critical emotional toll that childhood cancer can impose on families, warranting comprehensive support strategies that address parental mental health.

Moreover, our results indicate that lower educational levels among parents correlate with a higher likelihood of developing anxiety and depressive disorders. This finding aligns with Dąbkowska et al., who demonstrated that educational attainment is a significant factor influencing parental mental health in the context of childhood cancer [55]. This correlation may suggest that parents with lower educational levels face additional challenges in navigating the healthcare system, understanding medical information, and accessing support resources, which can exacerbate their emotional distress. Additionally, the duration of the child’s illness was found to significantly impact the severity of anxiety experienced by parents due to the long-term psychological burden associated with pro-longed treatment [19]. The chronic nature of the illness not only increases parental stress but also complicates their ability to maintain a sense of normalcy in family life. As parents grapple with their child’s ongoing treatment, they may also experience feelings of helplessness and uncertainty about the future, further contributing to their anxiety and depression [56]. Furthermore, the financial burden associated with childhood cancer treatment cannot be overlooked. One study highlighted that families often face significant out-of-pocket expenses, which can lead to financial strain and increased psychological distress [18]. Our findings support this notion, as we observed that financial concerns were a common source of anxiety among parents. The stress of managing medical costs, coupled with the emotional burden of their child’s illness, can create a compounded effect that significantly impacts their mental well-being. In light of these findings, it is evident that parents require robust psychological support systems to navigate the challenges associated with their child’s cancer diagnosis. Our research suggests that interventions should not only address the emotional needs of parents but also provide resources to alleviate financial burdens and enhance educational support. Programs that offer counseling services, peer support networks, and financial guidance could play a crucial role in improving the mental health outcomes of parents. In summary, this study highlights the urgent need for comprehensive psychological and social support interventions for parents of children with cancer. Addressing the identified emotional challenges is critical, as parental mental health directly influences the overall well-being and adaptation of the child to their illness. By tailoring support strategies that consider the relationships between anxiety, depression, and factors such as education and illness duration, we can better assist families in coping with the emotional difficulties associated with childhood cancer [57].

## 5. Limitations of This Study

The limitations of this study include the small sample size, which consisted of volunteers and may not be representative of the general population. Additionally, we only examined parents’ anxiety and depression, and other aspects of their distress were not assessed. Since we did not have access to the children’s medical records, most of the required data, such as medical treatment and the time elapsed since diagnosis, were collected based on the parents’ reports. Furthermore, most of our participants were mothers, who may have different emotional reactions than fathers. Previous studies indicate that mothers often experience higher levels of anxiety and depression when caring for a child with cancer, which can have a substantial impact on how they cope with the associated stress. This difference in emotional response can influence how parents cope with the stresses associated with their child’s illness [51,52].

## 6. Conclusions

Parents who experience severe anxiety are more likely to develop symptoms of depression. This finding underscores the need for psychological interventions that address both aspects. Moreover, a lower level of parental education significantly influences an increased tendency to develop depressive and anxiety disorders. Consequently, educational campaigns may have a key role in enhancing the ability to cope with the emotional burden associated with a child’s illness. Additionally, the duration of the child’s illness affects the severity of anxiety and depression in parents. A prolonged period of treatment and care for a sick child can lead to an increased emotional burden on parents.

Due to the significance of this topic, further research is necessary to explore the long-term psychological impact on parents of children with cancer, focusing on how coping strategies change over time. Additionally, future studies could investigate the effectiveness of targeted interventions aimed at reducing anxiety and depression in this population, considering variables such as gender and the duration of the child’s illness.

## Figures and Tables

**Table 1:** Descriptive statistics of the examined group of patients.

Demographic Information	Total N = 206	*p*
Characteristics % (N)		
Sex		
women	73% (150)	0.01
men	27% (56)	
Place of residence		
city	57% (117)	0.21
village	43% (89)	
Financial situation		
very good	18% (37)	0.01
good	55% (113)	
average	24% (50)	
bad	3% (6)	
Age groups		
18–24	5% (10)	0.01
25–29	16% (32)	
30–39	41% (84)	
40–49	35% (72)	
50 and above	4% (8)	
Education of the study group		
higher education	57% (117)	0.01
secondary education	36% (74)	
primary education	7% (15)	
Number of children in the family		
one child	35% (72)	0.71
two children	46% (95)	
three children	19% (39)	
Marital status		
married	62% (128)	0.62
informal relationship	19% (39)	
unmarried	19% (39)	

**Table 2:** Beck Depression Inventory results.

BDI	Descriptive Statistics						
	Mean	Median	Min.	Max.	Quartile I	Quartile III	Std. Dev.
[0–63 points]	20.63	18.50	0.00	63.00	12.00	27.00	12.39
Interpretation	Depression worsens						
	Points		% (N)			*p*	
no depression	From 0 to 11		23% (39)			0.12	
mild depression	From 12 to 19		33% (56)			0.01	
moderate depression	From 20 to 25		12% (20)			0.71	
severe depression	From 26 to 63		32% (55)			0.01	

**Table 3:** HADS-M scale results.

HADS-M	Descriptive Statistics						
	Mean	Median	Min.	Max.	Quartile I	Quartile III	Std. Dev.
Anxiety [pt]	10.17	10.00	1.00	21.00	7.00	14.00	4.36
Depression [pt]	9.45	10.00	0.00	21.00	7.00	12.00	4.79
Aggression [pt]	3.28	3.00	0.00	6.00	2.00	5.00	1.72
Anxiety [%]	48.43	47.62	4.76	100.00	33.33	66.67	20.78
Depression [%]	45.01	47.62	0.00	100.00	33.33	57.14	22.80
Aggression [%]	54.72	50.00	0.00	100.00	33.33	83.33	28.71

**Table 4:** Assessment of the relationship between the tendency for depression and the level of anxiety among parents.

Variables	R	*p*
Beck’s scale score and severity of depression in the HADS-M scale	0.68	<0.001
Beck’s scale score and severity of anxiety in the HADS-M scale	0.31	0.001
Beck’s scale score and severity of aggression in the HADS-M scale	0.58	<0.001

**Table 5:** The intensity of depression, anxiety, and aggression symptoms and variables.

Variables	Descriptive Statistics							
	Mean	Median	SD	Mean	Median	SD	Z	*p*
	Age of Respondents							
	Under 40			40 and over				
Depression (Beck)	20.82	18.00	12.22	20.34	19.00	12.80	0.23	0.815
Anxiety	46.08	42.86	20.31	52.15	47.62	21.21	−1.53	0.126
Depression	42.34	42.86	21.28	49.25	52.38	24.69	−1.41	0.157
Aggression	55.90	50.00	27.39	52.85	50.00	30.94	0.54	0.593
Gender								
	Women			Men				
Depression (Beck)	21.96	19.00	12.87	17.10	16.00	10.40	1.43	0.153
Anxiety	49.35	47.62	20.23	45.98	47.62	22.35	0.62	0.538
Depression	45.15	47.62	23.35	44.66	47.62	21.65	−0.12	0.901
Aggression	53.68	50.00	29.69	57.47	66.67	26.20	−0.68	0.494
Education								
	Secondary or lower			Higher				
Depression (Beck)	26.74	24.00	12.57	15.95	13.50	10.06	4.70	<0.001
Anxiety	54.04	54.76	19.98	44.13	45.24	20.50	2.34	0.019
Depression	53.52	57.14	19.68	38.49	42.86	23.02	3.53	<0.001
Aggression	57.25	58.33	28.25	52.78	50.00	29.14	0.68	0.495
Children’s age								
	Under 10 years			10 years and more				
Depression (Beck)	20.47	17.50	13.22	20.85	21.00	11.34	−0.63	0.526
Anxiety	45.08	45.24	22.00	52.80	47.62	18.40	−1.68	0.094
Depression	42.94	47.62	22.95	47.72	47.62	22.55	−0.71	0.477
Aggression	58.61	66.67	27.70	49.64	33.33	29.50	1.64	0.101
Duration of the child’s illness								
	Up to half a year			More than half a year				
Depression (Beck)	19.60	16.00	12.67	21.39	21.00	12.22	−0.97	0.330
Anxiety	40.74	38.10	21.81	54.10	52.38	18.15	−3.26	0.001
Depression	40.42	42.86	22.33	48.40	47.62	22.72	−1.52	0.127
Aggression	46.67	33.33	28.56	60.66	66.67	27.56	−2.52	0.012

SD—standard deviation.

## Data Availability

Data are available on request due to privacy and ethics restrictions.
